# Importance of early identification of PrEP breakthrough infections in a generalized HIV epidemic: a case report from a PrEP demonstration project in South Africa

**DOI:** 10.1186/s12879-020-05255-5

**Published:** 2020-07-22

**Authors:** Cherise L. Naicker, Leila E. Mansoor, Halima Dawood, Kogieleum Naidoo, Denzhe Singo, David Matten, Carolyn Williamson, Quarraisha Abdool Karim

**Affiliations:** 1 Centre for the AIDS Programme of Research in South Africa (CAPRISA), University of KwaZulu-Natal, Durban, South Africa; 2 MRC-CAPRISA HIV-TB Pathogenesis and Treatment Research Unit, University of KwaZulu-Natal, Durban, South Africa; 3 Division of Medical Virology, University of Cape Town, Cape Town, South Africa; 4 The National Health Laboratory Service, Cape Town, South Africa; 5 Department of Epidemiology, Mailman School of Public Health, Columbia University, New York, USA

**Keywords:** HIV prevention, Pre-exposure prophylaxis (PrEP), Tenofovir, PrEP breakthrough infections in HIV-endemic settings, Fourth-generation rapid tests for HIV screening, HIV drug resistance

## Abstract

**Background:**

The World Health Organisation recommends the use of tenofovir-containing pre-exposure prophylaxis (PrEP) as an additional Human Immunodeficiency Virus (HIV) prevention choice for men and women at substantial risk of HIV infection. PrEP could fill an important HIV prevention gap, especially for sexually active young women who are limited in their ability to negotiate mutual monogamy or condom use.

As PrEP is scaled up in high HIV incidence settings, it is crucial to consider the importance of early identification of HIV infection during PrEP use, to allow for rapid discontinuation of PrEP to reduce the risk of antiretroviral (ARV) resistance. The purpose of this case study is to provide this critical evidence.

**Case presentation:**

This report describes a 20-year-old woman in a HIV sero-discordant relationship who initiated oral PrEP (tenofovir disoproxil fumarate (TDF) and emtricitabine (FTC)) through a demonstration project (CAPRISA 084) in October 2017. Despite good adherence throughout her PrEP use, she tested HIV antibody positive at month nine of study participation.

Retrospective testing showed increasing HIV viral load over time, and retrospective use of fourth-generation rapid HIV tests showed HIV detection (positive antigen/antibody) at month one. Sequencing confirmed a dominant wild type at month one with dual therapy resistance patterns emerging by month three (M184V and K65R mutations), which is suggestive of protracted PrEP use during an undetected HIV infection.

The participant was referred to infectious diseases for further management of her HIV infection and was initiated on a first line, tenofovir-sparing regimen. At the time of this report (January 2020), the participant had been on ARV- therapy (ART) for 13 months and had no signs of either clinical, immunologic or virologic failure.

**Conclusions:**

This case report highlights the importance of appropriate HIV screening during wider oral PrEP scale-up in high HIV incidence settings to circumvent the consequences of prolonged dual therapy in an undiagnosed HIV infection and in turn prevent ARV resistance.

## Background

The World Health Organisation (WHO) 2015 antiretroviral (ARV) guidelines recommend the use of tenofovir (TFV) containing regimens for pre-exposure prophylaxis (PrEP) as an additional Human Immunodeficiency Virus (HIV) prevention tool for those at risk of acquiring HIV, specifically in high HIV incidence settings [1]. This has led to oral PrEP being incorporated as part of a comprehensive HIV prevention package in several countries, including South Africa [2, 3]. In Africa, a key target for PrEP implementation is young women, the group at highest risk [1]. Several PrEP demonstration projects are underway to inform programmatic scale-up of oral PrEP [2].

CAPRISA 084, a PrEP Demonstration Project, assessed the feasibility, acceptability, uptake and patterns of daily oral tenofovir disoproxil fumarate (TDF) / emtricitabine (FTC) use as part of sexual reproductive health services to young women and men at risk of acquiring HIV in urban and rural KwaZulu-Natal, South Africa. The project was conducted between August 2016 and September 2018. The protocol, informed consent forms and study related materials were reviewed and approved by the University of KwaZulu-Natal’s Biomedical Research Ethics Committee (Ref: BFC511/16). All participants provided written informed consent. Follow-up of enrolled participants occurred monthly for 3 months post-PrEP initiation and quarterly thereafter. Clinical evaluation, adherence counselling and rapid HIV testing using two third-generation HIV antibody tests (Uni-Gold Recombigen HIV-1/2 and Alere Determine HIV-1/2 (Alere)), were conducted at screening, enrolment and at each follow-up visit. Dry blood spots (DBS) were collected and stored from month one onwards. Screening for sexually transmitted infections (STIs) occurred quarterly.

## Case presentation

### Clinical summary

We report clinical data from a 20-year-old woman, in a HIV sero-discordant relationship, who was initiated on daily oral PrEP in October 2017 through CAPRISA 084 at an urban site in South Africa. The participant attended the study clinic regularly and clinical presentation at study visits was unremarkable, with no report of seroconversion illness. Self-reported PrEP adherence, including pill count, was high. She was diagnosed with HIV infection, using two third-generation HIV rapid antibody tests, 9 months after initiating daily oral PrEP (July 2018), with a HIV viral load of 31,730 copies/ml. Prolonged inadvertent dual therapy during an undetected HIV infection resulted in nucleoside reverse transcriptase inhibitor (NRTI) mutations to both TDF and FTC. The participant was referred to a specialist infectious diseases clinic for further management of her HIV infection. She was initiated on a first line, TFV-sparing regimen (zidovudine (AZT) / lamivudine (3TC) / efavirenz (EFV)) in October 2018. Her month five HIV viral load (March 2019) was 380 copies/ml. At the time of this report (January 2020), the participant had been on ART for 13 months and had no signs of either clinical, immunologic or virologic failure on her treatment regimen. The postulate for HIV acquisition is: (i) recent occurrence of HIV infection prior to oral PrEP initiation, (ii) HIV exposure early in her PrEP use before adequate oral PrEP drug levels were achieved, and (iii) acquisition of a resistant viral strain from her sexual partner.

### Confirmation of HIV-1 infection

**Fig. 1:**
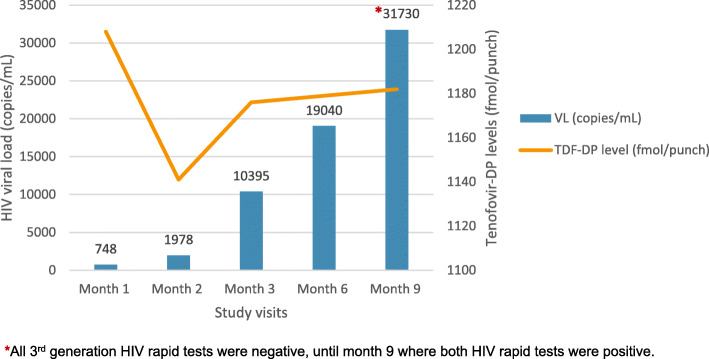
Participant HIV viral load and TDF-DP levels at different time-points in study participation

HIV seroconversion was confirmed by HIV-1 GEENIUS assay and HIV-1 viral load testing (Roche Ampliprep-Taqman assay) at the month nine visit. Retrospective HIV viral load testing was done on all available stored samples which showed an increasing HIV viral load trend from month one onwards (Fig. 1). In addition, retrospective HIV testing using fourth-generation test kits (Alere HIV Combo), which detects antigen and antibody, was conducted on available stored samples which revealed HIV detection from month one onwards. In addition, all participants remaining in the CAPRISA 084 study were tested with the fourth-generation HIV rapid test, in addition to the third-generation HIV antibody tests, to exclude any additional masked HIV infections.

### Drug level monitoring

Tenofovir-diphosphate (TDF-DP) levels were retrospectively measured from DBS samples, from every visit, using a modified liquid chromatography tandem mass spectrometry assay [4]. This validated assay (Division of Clinical Pharmacology, University of Cape Town), was conducted in batches by a trained technologist. Given that drug level measurements were not conducted in real-time they were not used for clinical management. Her TDF-DP levels ranged between 1100 and 1200 fmol/punch, corresponding to daily dosing (6–7 tablets per week) of oral PrEP (Fig. 1).

### HIV drug resistance and deep sequencing testing

**Fig. 2:**
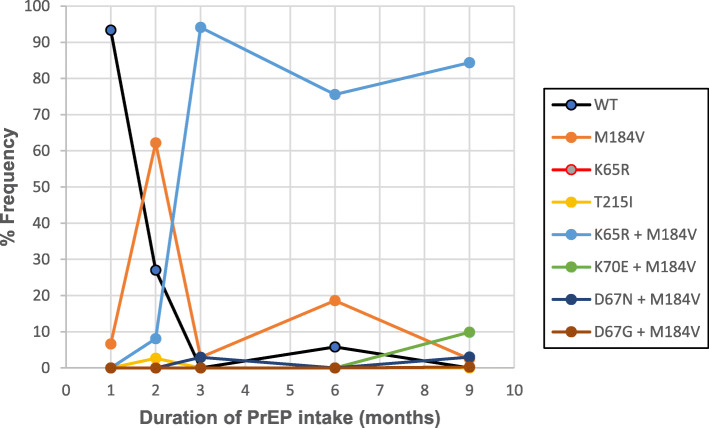
Results from HIV deep sequencing demonstrating total percentage frequency of NRTI resistance mutations

HIV drug resistance testing was done at the highest HIV viral load time-point (month nine sample) using the SATuRN/Life Technologies genotyping system, using four sequencing primers to generate the 1197 bp *pol* sequence covering all 99 HIV-1 protease codons and the first 300 codons of the reverse transcriptase gene [5]. Amplification and deep sequencing of the *pol* gene (reverse transcriptase region = HXB2 2735–3244) using the Illumina MiSeq platform using primer ID approach for quantification [6] was carried out on all available DBS samples, including those with low HIV viral loads, from month one onwards (Fig. 2). Deep sequencing was undertaken to ascertain whether the drug mutations found in the participant were transmitted from her partner or caused by drug pressure from oral PrEP.

HIV drug resistance testing on the participant’s month nine sample revealed the presence of both the M184V and the K65R mutations. The M184V mutation is linked to FTC and 3TC high level resistance, whilst increasing susceptibility to thymidine analogue NRTIs [7]. This mutation also decreases HIV-1 replication capacity. K65R mutation is the signature TFV resistance mutation and confers intermediate/high-level resistance to TDF, didanosine (ddI), abacavir (ABC) and stavudine (D4T) and low/intermediate resistance to 3TC and FTC. This mutation also results in increased susceptibility to AZT [7]. HIV drug resistance testing showed none of the following: protease inhibitor major, protease inhibitor minor, non-NRTI, integrase inhibitor major and integrase inhibitor minor resistance mutations.

Results of deep sequencing of the virus showed dominant wild type virus with no drug resistance mutations (< 5%) at month one. However, by 2 months the M184V mutation gained selective advantage and became dominant, and by 3 months, dual resistance was observed with the detection of the K65R mutations with nearly 100% resistant viral population (Fig. 2).

### Elimination of transmitted ARV drug resistance

HIV viral load testing was carried out on the participant’s partner at the time of diagnosis of HIV infection in the participant. The sample was retained for HIV drug resistance testing, to elicit any ARV drug mutations present in the partner, if required. However, HIV drug resistance testing for the partner was not performed as he had an undetectable HIV viral load on first line ART (TDF/FTC/EFV) at the time of diagnosis of HIV infection in the participant. In addition, the partner had an undetectable viral load at both 6- and 12-months post-ART initiation.

## Discussion and conclusion

This case reports an oral PrEP breakthrough infection despite confirmed high adherence to PrEP in a 20-year-old woman who was found to be HIV infected 9 months after PrEP initiation. A key first step is ensuring that PrEP is being initiated in an HIV uninfected person to minimise a recently infected person being initiated on dual therapy. Equally important is counselling on safer sex practices in the first month of PrEP initiation.

No stored samples were available from the participants’ screening and enrolment visits; hence a window period of HIV infection may have existed and cannot be excluded.

A review of oral PrEP related studies revealed an implementation study where a 41% decrease in condom use amongst a subset of PrEP users was found [8, 9]. Despite this, no new HIV infections among PrEP users were observed [9]. Safer sex practices, especially in the first 20 days for vaginal receptive sex whilst PrEP drug levels are reaching steady state concentration, needs special attention.

PrEP efficacy trials indicate that cases of acquisition of ARV resistance are infrequent, predominately limited to individuals receiving PrEP around the time of HIV seroconversion. Furthermore, prolonged PrEP use after HIV infection also promotes acquisition of resistance [10, 11]. This is due either to infrequent follow-up or poor performance of available HIV rapid tests in the setting of PrEP use [12, 13]. Failure to detect HIV infection using third generation rapid tests has been previously reported and resulted in continued PrEP use in eight cases [14]. This finding emphasizes the importance of careful HIV screening especially in the setting of wider oral PrEP scale-up in HIV endemic settings where PrEP-related resistance may limit options for subsequent ART.

PrEP-selected resistance (M184V mutation) is strongly selected by FTC and 3TC and emerges rapidly in patients receiving either of these drugs. Also, the M184V mutation occurs more frequently with dual ART use rather than triple therapy [15]. The M184V mutation virus strain adversely affects viral replicative capacity and is less fit compared to the wild type strain, hence it is less likely to be transmitted during infection [16]. Also, the undetectable HIV viral load at both 6- and 12-months post-ART initiation in the partner further supports that the noted drug mutations in the participant were PrEP-induced rather than transmitted from her partner.

The findings of the HIV deep sequencing (i.e. dominant wild type virus at month one) suggest that resistance developed as a result of PrEP use, as one would expect that M184V would be a major population from the outset if it was a transmitted virus. The low genetic barrier brought about by using a combination of only two drugs (TDF/FTC) resulted in the emergence of other key mutations selected by TDF, e.g. K65R which we see in the later time points.

PrEP-selected resistance is more likely in the presence of adequate drug pressure, since PrEP acted as an inadequate ART regimen, i.e. dual therapy, however, intermittent PrEP use can result in low and fluctuating drug levels, an environment in which selective pressure for resistance is periodic, and thus resistant mutations could be present at only low frequencies [17]. Conversely, in the presence of poor PrEP adherence the risk of drug resistance is far less due to the lack of drug pressure [17, 18].

Because it is unlikely that PrEP will prove 100% efficacious, it is critical to understand the impact of PrEP on the immune response to HIV for individuals who become infected despite PrEP use. Possible delay in detection of HIV-1-infection as a result of PrEP use would be a concern if the recommended quarterly HIV testing missed diagnoses and inadvertently prolonged PrEP exposure after infection, thereby increasing risk of resistance mutations.

Some studies have demonstrated blunted acute viraemia in PrEP breakthrough infections and likely reflects the strong ARV activity by the PrEP regimen [19–21]. Subsequently, the attenuated HIV viral load associated with ARV use alters antibody production by decreasing titres or preventing antibody responses to certain viral antigens, possibly as a result of the paucity of adequate antigenic exposure [22]. Similarly, in a study of rhesus macaques, PrEP was associated with delayed antibody maturation [23]. This delayed antibody avidity raises the concern that PrEP may affect the performance of assays used to detect HIV infection in the setting of PrEP use.

The findings of this case report support the above conclusions that prolonged use of oral PrEP during undetected HIV infection may reduce clinical symptoms of HIV infection and be associated with delayed antibody avidity, as well as sustained partial viral suppression, which would in turn make it difficult to diagnose HIV infection, particularly if less specific assays are used for HIV screening, such as third-generation HIV rapid tests. A potential increase in acquisition of PrEP-related resistance would jeopardize choice of ART for both the PrEP user and their partner/s. However, the risk of potential PrEP-related resistance needs to be weighed against the number of HIV infections averted. The few documented cases of true PrEP-selected resistance could suggest that resistance is likely to be rare as PrEP is scaled up [16, 24–28]. Another trend seen on drug level testing is that the majority of seroconverters in PrEP studies were non-adherent to their oral PrEP at the time of seroconversion, resulting in low risk of resistance because of the lack of drug pressure [18].

During scale up of oral PrEP in South Africa, it is crucial to consider the importance of accurate identification of HIV infection prior to oral PrEP initiation and rapid discontinuation of PrEP in those suspected to be HIV infected to reduce the risk of HIV drug resistance. This case provides evidence that support a recommendation for the use of fourth-generation HIV rapid tests for routine HIV screening in the setting of PrEP use and highlights the importance of prompt ARV resistance testing once HIV infection is identified, initiation on the appropriate ART regimen and subsequent follow-up of both participant and partner/s for early detection of ART regimen failure. This case also demonstrates that despite the prolonged exposure to dual therapy, if the above prompt actions are taken, HIV virological suppression is achievable with a first line ART regimen.

## Data Availability

The datasets used and/or analysed during the current study are available from the corresponding author on reasonable request.

## Abbreviations

ABC: Abacavir
ART: Anti-retroviral therapy
ARV: Anti-retroviral
AZT: Zidovudine
ddI: Didanosine
D4T: Stavudine
DBS: Dried blood spots
EFV: Efavirenz
FTC: Emtricitabine
HIV: Human Immunodeficiency Virus
NRTI: Non-nucleoside reverse transcriptase inhibitor
PrEP: Pre-exposure Prophylaxis
STI: Sexually transmitted infections
TDF: Tenofovir disoproxil fumarate
TDF-DP: Tenofovir diphosphate
TFV: Tenofovir
WHO: World Health Organisation
3TC: Lamivudine
